# Authors’ Response to Peer Reviews of “Information Technology Ambidexterity, Digital Dynamic Capability, and Knowledge Processes as Enablers of Patient Agility: Empirical Study”

**DOI:** 10.2196/34106

**Published:** 2021-12-06

**Authors:** Rogier van de Wetering, Johan Versendaal

**Affiliations:** 1 Department of Information Sciences Open University of the Netherlands Heerlen Netherlands

**Keywords:** IT ambidexterity, dynamic capabilities, digital dynamic capability, knowledge processes, patient agility, hospitals, information sciences, information technology, digital health, health care, digital transformation, research models

*This is the authors’ response to peer-review reports for “Information Technology Ambidexterity, Digital Dynamic Capability, and Knowledge Processes as Enablers of Patient Agility: Empirical Study”*

## Round 1 Review

Comments and Responses by the Authors [1]

### Reviewer AE

1. Methods
  - Describe the study [1] settings
  - Move the highlighted (in the reviewed manuscript) content under Data Collection Procedures to a new subsection under subsection heading Study Population (see comments in the reviewed manuscript)
  - The highlighted content should be under a new subsection heading Study Design
  - Provide content on another two subsection headings:
    - Sampling Techniques
    - Sample Size
  - Separate content under Data Collection Procedure into two new subsection headings
    - Data Collection Tool and Procedure
    - Data Analysis and Management
  - Move Table 1 to Analyses & Results section
  - Provide content under two new subsection headings:
    - Inclusion and Exclusion Criteria
    - Ethics Considerations

Authors: The sections mentioned in this review [2] are essential, and therefore, we added them to the article’s Methods section. Concerning Ethics Consideration, respondents were allowed to complete the survey anonymously, and we did not log anything in the survey system that could trace respondents. Also, reusable personal data was not requested, and the survey did not include questions about personal or sensitive topics. Furthermore, before starting the survey, the respondents had to sign a consent form. This approach is in line with the General Data Protection Regulation. Finally, respondents were given the option to leave their email addresses to receive a research report. These email addresses were removed from the data set after this report was sent.

Inclusion and exclusion criteria were combined with sample size, as we described how we got the final sample and why we included respondents (and why not).

### Minor Comments

1. Abstract
  - Do not begin a sentence with abbreviation of figure
  - Use past tense under Methods (eg, consider ‘used’ in lieu of ‘uses’)
  - See comments in the reviewed manuscript
2. Introduction
  - Use physicians in lieu of doctors
  - Use health care providers not other medical professionals
  - Keep in-text citation to the end of sentence
  - Add health information management professionals among the key stakeholders
  - Consider reducing the whole of section 2 (Theoretical Background) to 1-2 paragraphs and keep it within the Introduction section just before your study objective. This is to reduce readers’ boredom.
  - Compress the content under research models and hypotheses
3. Results
  - Make your findings more visible here
  - Make your writing more readable to known and unknown readers
4. Discussion
  - Plausible and insightful discussion but not a reflection of the content under the Results. Make the Results section more readable and meaningful to your audience.
5. Figure
  - Use Fig not Figure
6. Acknowledgement
  - It is scientifically necessary that you acknowledge the numerous (n=107) participants, who are the major stakeholders in your research.
7. Reference
  - List at least 3 authors before et al
  - Follow the Referencing Style consistently
8. Others
  - Use participants not respondents

Authors: We adjusted this accordingly.

### Reviewer AO

#### General Comments

Thank you for the opportunity to review this paper on the lesser known topic of information and communications technology ambidexterity. The paper is well cited, uses appropriate methods, and discusses the concepts and findings in a clear and thorough manner. The paper should appeal to a broad audience. It is a good example of the underrepresented information and communications technology–centered literature in health care.

Authors: We would like to thank this reviewer for these kind words [3]. We hope that we can make a great contribution to this journal and the field.

### Reviewer BQ

#### General Comments

Well thought out study design with specific hypotheses and methods of analysis spelled out. Interesting conclusions drawn out that would be fruitful for further discussion and analysis to replicate on a broader sample of hospital systems outside of the current reviewed sites.

Authors: We would also like to thank this reviewer for these kind words [4]. We agree that there could be a very interesting follow-up study.
